# Perception of Cognitive Functions and Academic Performance in Chilean Public Schools

**DOI:** 10.3390/bs12100356

**Published:** 2022-09-24

**Authors:** Jacqueline Valdebenito-Villalobos, María Antonia Parra-Rizo, Yasna Chávez-Castillo, Caterin Díaz-Vargas, Gloria Sanzana Vallejos, Aurora Gutiérrez Echavarría, Andrea Tapia Figueroa, Xeny Godoy Montecinos, Rafael Zapata-Lamana, Igor Cigarroa

**Affiliations:** 1 Escuela de Educación, Universidad de Concepción, Los Ángeles 4440000, Chile; 2 Faculty of Health Sciences, Valencian International University(VIU), 46002 Valencia, Spain; 3 Department of Health Psychology, Faculty of Social and Health Sciences, Campus of Elche, Miguel Hernández University (UMH), 03202 Elche, Spain; 4 Grupo Interdisciplinario en Educación GIIE, Escuela de Educación, Universidad de Concepción, Los Ángeles 4440000, Chile; 5 Facultad Ciencias Sociales, Estudiante de Doctorado en Psicología, Universidad de Concepción, Concepción 4030000, Chile; 6 Facultad de Educación, Universidad de Concepción, Concepción 4030000, Chile; 7 Escuela de Kinesiología, Facultad de Salud, Universidad Santo Tomás, Los Ángeles 4440000, Chile

**Keywords:** cognitive processes, academic performance, self-perception, cross-sectional design, Chile

## Abstract

Understanding the perception that students have about their own cognitive processes is a key aspect that allows for a deeper assimilation of the different factors that affect school performance. However, there is limited evidence explaining the link between students’ perception of their own cognitive functions and their academic performance. The objective of this study was to analyze the association between perception of cognitive functions, such as memory, processing speed, attention, execution of complex tasks and nervousness, and academic performance in Chilean schoolchildren from the province of Biobio. A cross-sectional analytic design was conducted. The sample consisted of 590 elementary school students (12 ± 1.3 years old; 48.3% female) from Chilean public schools. The academic performance was measured by means of the accumulated final grades in the language, mathematics, physical education and health subjects, and the grade point average (GPA) of each student. Moreover, a survey to measure the cognitive functions of the participants was applied. The results show that 20.3% of the students perceived themselves as very nervous and 16.8% felt distracted. Differences in marks were observed in all the measured subjects, as well as in GPAs, depending on the perception of cognitive functions. Thus, the students with low to moderate perceptions of their cognitive functions received lower marks than those who reported a high perception. These results were consistent after a multivariate analysis adjusted for a socio-educational variables model. In conclusion, one in five public school students in the Biobío Region of Chile expressed a low perception of their cognitive functions, which is consistent with their weaker school performance. Therefore, it is believed that integrating curricular activity and cognitive work could potentially boost the perception of said functions, and thus reduce the risk of poor academic performance.

## 1. Introduction

The study of school performance has always been an interesting focus of attention in education. Plenty of research has been carried out around this topic, considering several variables and related aspects, with the purpose of answering a question that, even with efforts form the public policies, does not seem to find an accurate answer. In this sense, understanding the perception that students have of their own cognitive processes seems to be an interesting area of study, aimed at understanding how they perceive their learning and assess their cognitive performance in terms of certain variables that are directly related to their academic achievement.

Moreover, academic performance is a multidimensional concept, which includes cognitive, educational, family, and environmental factors [1,2,3,4]. Since academic performance is considered a key aspect in the teaching-learning process [5], the interaction between the different factors that influence it, and their association to success, failure and desertion of studies has been deeply studied [2]. Furthermore, academic performance is seen as an indicator of quality of education, which must be reached by students, teachers, and the assessment system as a whole [6]. It is expressed as a mark; an accurate and accessible quantitative indicator that certifies the level of attainment reached by the students [7], reflecting the level of academic performance in the different components of the learning process [2,8]. Academic performance is of such important that Joseph et al. (2014) state that it is closely related to job performance; a higher academic performance could potentially predict a successful job performance [9].

“Low-Performing Students: Why they fall behind and how to help them succeed”, a report by the OECD, states that students with low academic performance display dismissive attitudes towards school and learning in general, a low index of perseverance, low motivation, and low self-confidence, along with less support from their teachers and schools [10]. Chilean students’ results in the international test program for international student assessment show that 68% of students presented low performance in reading (OECD average: 9%) and 48% presented low performance in mathematics (OECD average: 76%) [11]. In the past years, Chile has reduced the number of students with low academic performance, however, there are still challenges that persist, such as the study of the students’ perception about cognitive functions and their relationship with learning.

Among the factors that define academic performance, it is possible to find higher order cognitive functions which regulate the necessary behavior, emotions, and cognition for reaching goals, solving problems, perform superficially learned actions, and adapting to new or complex situations [12], such as memory, processing speed, attention, complex task execution skills and nervousness. In this sense, Garbanzo G. (2013) points out the importance of studying the cognitive variables associated with academic performance beyond marks, which would allow improving the quality of education [7].

Additionally, memory refers to the processes that hold and transform information in short periods of time [13], which must be then consolidated in the long-term memory to achieve learning. This ability plays a crucial role in academic achievement throughout school years. Several current studies have pointed out that a short memory is a relevant predictor of academic performance, even more relevant than intellectual quotient [14], due to its impact in reading comprehension, arithmetic competences, and necessary behavior for learning.

Regarding processing speed, research in the area by Diaz J. et al. (2018) evidenced challenges that students face when solving math problems, for instance, lack of understanding which does not allow for an appropriate solution search, as well as incoherent answers, unwillingness to solve problems as a product of previous negative experiences, lack of mental processes regulation, and time constraints, which does not encourage reflection [15].

Furthermore, attention is a mechanism that allows stimuli, thought and action processing, as well as resisting distractions and irrelevant information [16]. It encompasses a series of cognitive processes, such as relaxing attentional focus, controlling interference, detecting mistakes, and distributing additional attentional resources. According to Gaulin C. (2001), complex task execution involves contemplating situations that demand reflection, search, and research in which thinking about solutions and defining strategies do not necessarily provide an immediate answer [17]. Finally, as Martínez E. et al. (2007) state, nervousness is associated with school stress, which produces tension, tiredness, strain, and restlessness as a response to academic pressure [18].

There is a lack of evidence that has analyzed the perception of the cognitive functions of the students. According to what was studied, there is no study in South American countries that has focused on relating the perception of cognitive functions and academic performance in schoolchildren. Based on this background, the research question arises:

Is there an association between the perception of cognitive functions, such as memory, processing speed, attention, execution of complex tasks and nervousness, and academic performance in Chilean schoolchildren?

The objective of this study was to analyze the association between perception of cognitive functions, such as memory, processing speed, attention, execution of complex tasks and nervousness, and academic performance in Chilean schoolchildren from the province of Biobio.

## 2. Materials and Method

### 2.1. Design

Analytic cross-sectional study in which data from the 2018 national well-being and academic performance survey in the Biobío province were used [19].

### 2.2. Participants

The participants were fifth grade to eight grade students from all the public schools from one of the districts in the Biobío province (*n* = 3857). A stratified, probabilistic and community representative sample of 797 Chilean students (12 ± 1.3 years) was included. These students participated after their parents or legal tutors had signed the informed consent. A total of 64 students were excluded from the study since they were not present during the day of the measurements and their parents or legal tutors not signing the informed consent, and 143 students were excluded due to being part of the school inclusion program. The final sample included 590 students (51.7% male; 12 ± 1.3 years). The data analysis contemplates a 5% margin of error and a 95% confidence interval.

### 2.3. Procedure

An Alliance between the research team and the district’s education department (DAEM) was formed. Once the approval of the ethics committee and the municipal permission were granted, the design of the study and the selection of variables were made in conjunction with the schools’ boards. Then, the teachers involved were trained in the application of the instruments in order to reduce inter-rater bias. Consequently, the data gathering process was carried out during the same period, on the same day in all the schools.

The families, principals, and teachers of all the schools were informed about the nature and purpose of the study through a document, which was read and signed by all of them, to collaborate with the project. The study was carried out following the ethical, legal, and regulatory framework for human subjects research. The study protocol was approved by the Bioethics and biodiversity committee of the Universidad de Concepción, Nº 1.0-RZL-Abril/2018, and all the procedures were carried out according to the Declaration of Helsinki for research with human subjects.

### 2.4. Variables and Instruments

*Academic performance:* This was measured through the accumulated final grades in the subjects of language (Spanish), mathematics, physical education and health, as well as the grade point average (GPA) reached during the first academic semester. The marks range from 1.0 to 7.0, where 4.0 is the minimum passing mark. No differences in academic requirements were considered since all schools are public and are regulated by the same curriculum provided by the Ministry of Education [20,21]

*Perception of cognitive functions in educational contexts:* a survey designed by the research team, including 5 out of 18 items from the scale on daily stress [22] was applied. These items are associated with the perception of cognitive functions in educational contexts, and have been used in other studies [23,24]. The 5 questions included were: How good is your memory? How fast can you solve a math problem? How well can you focus during classes without getting distracted? How well can you execute complex tasks in school? How nervous do you get during tests? Each question was graded from 0 to 10, where the lowest values indicated problems with the evaluated behaviors, and the highest values represented better behaviors. Then, to present the results, three levels were proposed for each behavior (0–3, 4–6 and 7–10). Thus, the levels were: (a) Memory in classes: poor memory (0–3), moderate memory (4–6), and good memory (7–10); (b) Processing speed: low speed (0–3), moderate speed (4–6), and fast speed (7–10); Attention in classes: poor attention (0–3) moderate attention (4–6) and good attention (7–10); (d) Complex task execution: It costs me great effort (0–3), I have some problems (4–6), and I have no problems (7–10); and finally nervousness during tests: Very nervous (0–3), somewhat nervous (4–6) and relaxed (7–10).

### 2.5. Socio-Educational Data

Additionally, other factors such as sex, age, grade of each student, and school according to their level of performance were reported. These aspects are important components of the education quality assurance system, which, through integral assessment, classifies schools in high, medium, low-medium and insufficient performance [25].

### 2.6. Statistical Analysis

The data were analyzed by means of the statistical software SPSS 25.0 (IBM SPSS statistics, Chicago, IL, USA). A descriptive analysis including socio-educational (age, sex, grade and school) and academic performance (GPA and perception of cognitive functions in educational contexts) variables was carried out. The qualitative data were represented through absolute and percentage frequency, while quantitative data were represented through the mean and its corresponding 95% confidence interval. The distribution of data was analyzed through a Kolmogorov-Smirnoff test and the equality of variances through a Levene test, which showed a normal distribution and homogeneity of variances, thus leading to a parametric statistical analysis. To present the results, for each behavior a category was stablished (0–3, 4–6, 7–10). Thus, the categories were: (a) Memory in classes: poor memory (0–3), moderate memory (4–6), good memory (7–10); (b) processing speed: low speed (0–3), moderate speed (4–6), fast speed (7–10); (c) attention in classes: poor attention (0–3), moderate attention (4–6), good attention (7–10); (d) complex task execution: It costs me great effort (0–3), I have some problems (4–6), I have no problems (7–10); (e) nervousness during tests: Very nervous (0–3), somewhat nervous (4–6), relaxed (7–10). A one-way ANOVA test was used. In the case of significant differences, a post hoc test (Bonferroni) was used to confirm where the differences occurred between the groups. A multiple linear regression analysis was used for analyzing the association between the GPAs and the perception of cognitive functions. The data were expressed as β level with its corresponding 95% confidence interval. The multivariate model was adjusted for relevant confounding variables, selected from socio-educational characteristics. Model 0: non adjusted and Model 1: adjusted for socio-educational variables (age, sex, grade and school). Significance level *p* < 0.05.

## 3. Results

Table 1 shows socio-educational characteristics, as well as final accumulated marks in the subjects of mathematics, language (Spanish), physical education and health, and the students’ GPAs. It was possible to observe that most participants were male (51.7%), between 12 and 13 years of age. When comparing final accumulated marks in the mentioned subjects, it was also observed that girls achieved better marks in language than boys. Girls also had better GPAs than boys. Furthermore, it was evident that students between 11 and 12 years of age achieved better marks in mathematics and physical education and health, as well as better GPAs than girls.

Table 2 displays perception of cognitive functions in educational contexts. It was observed that most students considered they had good memory (63.6%), were fast to solve math problems (54.7%), could pay moderate attention (41.3%), had no problems to execute complex tasks (50.1%), and were relaxed while taking tests (54.4%).

In Table 3, academic performance according to perception of cognitive functions in educational contexts is illustrated. The ANOVA analysis reported significant differences in the final accumulated grades per subject in mathematics, language (Spanish), physical education and health, as well as GPAs, according to the perception of cognitive functions in the educational context (memory in classes, processing speed, attention, and complex task execution). After a deeper analysis (post hoc Bonferroni) of the students’ perception of their memory, it was observed that those who perceived themselves as having poor memory attained lower marks in language (Spanish), as opposed to those who perceived themselves as having good memory. Moreover, the evidence showed that those students who perceived themselves as having moderate and bad memory attained lower GPAs than those who described their memory as good (Table 3).

In terms of processing speed, it was detected that those students who perceived their speed as low or moderate achieved lower accumulated marks in language (Spanish) and GPAs, as opposed to those self-perceived with fast processing speed. In addition to this, it was observed that those students who perceived their processing speed as low also achieved lower final accumulated marks in mathematics than those who classified their speed as moderate. The latter also presented lower marks than those self-perceived as fast (Table 3).

Regarding the attention in classes, it was noticed that the students who labeled theirs as poor or moderate attained lower accumulated marks in mathematics, language (Spanish), physical education and health, and GPAs in contrast with those who reported good attention. In the matter of complex task execution, the students who expressed having to make great effort or having some problems obtained inferior accumulated marks in mathematics and language, and GPAs, contrary to those who reported having no problems when facing this type of task. Finally, in terms of nervousness during tests, it was detected that the students who reported being somewhat nervous or relaxed achieved better GPAs than those who perceived themselves as very nervous (Table 3).

Table 4 portrays the relationship between marks and perception of cognitive functions in educational contexts. In the non-adjusted model, the students who perceived themselves having poor and moderate memory scored lower marks in language as well as getting a lower GPA, in contrast with those who perceived themselves as having good memory. Likewise, the students who reported having moderate memory attained inferior marks in language and physical education and health than those students who indicated having good memory. Regarding processing speed, it was attended that the lowest marks were obtained by the students who had expressed low to moderate speed in all subjects and GPAs. On top of that, students who reported poor or moderate attention and several to some problems at executing complex tasks received lower marks in mathematics, language, and GPAs, as opposed to those reporting high processing speed and no problems when executing complex tasks. Finally, the students who specified being very nervous during tests attained lower marks in physical education and health and a lower GPA as contrasted with those who expressed feeling relaxed. Similar results were obtained in a model adjusted for socio-educational confounding variables (sex, age, grade, and school). Concerning the perception of nervousness, these results were not consistent when adjusting the model for socio-educational variables (Table 4).

## 4. Discussion

### 4.1. Main Results of This Study

The main results of this study point out that marks in mathematics, language (Spanish)*,* physical education and health, as well as GPAs are different according to sex, age, grade, and the schools’ level of performance. 20.3% of the students perceived themselves as very nervous during tests and 16.8% reported poor attention. Moreover, it was detected that the students who presented low to moderate perceptions of their cognitive functions received lower marks, as opposed to those who reported high perceptions. These results were independent of confounding variables such as sex, age, grade, and schools’ level of performance.

### 4.2. How the Results Can Be Interpreted in Perspective of Previous Studies?

Firstly, differences in academic performance according to sex, age, grade, and the schools’ level of performance were observed. These results are consistent with the existing literature [1,26,27,28]. For instance, a Spanish study with a sample of 535 students between 7 and 11 years old, showed that not only attitude, but also interest in schoolwork tends to decrease as the course progresses, which directly influences academic performance [29]. Thus, the results show that girls achieved better scores than boys in all of the evaluated courses, particularly in reading.

Regarding the perception of cognitive functions, it was noticed that around 20% of the students perceived themselves as very nervous during tests, while 16.8% reported low levels of attention in classes. In this context, and consistent with our results, Alfonso et al. (2015) consider academic stress as a wide range of experiences, such as nervousness among others [30], while Trianes M, (2003) states that schools are spaces in which several stressful situations for students can be generated [31]. For instance, in a study from a sample of 405 Spanish secondary education students, a 43.3% of students who perceived themselves nervous while taking tests, and a 22% who reported poor skills for managing stressful situations, such as evaluations [32]. These results are also consistent with a study in which 7808 Spanish students expressed that one of their biggest fears was “getting low marks”. Additionally, attention is associated with high academic performance [33], allowing the students to activate, direct, and control actions to reach objectives through positive stimuli [34], hence the importance of designing activities and programs to improve attention levels, due to its pivotal role in learning.

In the same way, the nervousness reported by the students can be a product of educational or family matters, since dysfunctionality in the family and pressure in the school context, which often focuses on results rather than the learning process, neglect socio-emotional aspects that are essential when working with students. Furthermore, in the context of family matters, a recent study state that dysfunctionality issues, such as family disintegration, parenting styles, lack of interest from the parents, substance abuse, favored or unwanted children, could trigger nervousness and academic stress [35]. Accordingly, Lastre et al. (2017) point out that accompaniment, presence, and dedication from the families are key, not only to achieve high academic performance, but also for emotional training [36]. In regard to educational contexts, it is important to mention that despite the efforts made from 2016 onwards, when a new system focusing on a balance of instruments and assessment techniques was implemented, including a wide range of formative and summative assessment instances [37], it is still possible to detect the pressure the students go through, due to the emphasis that is placed on content coverage and learning, often measured through repetitive standardized tests.

In terms of the low perception of attention reported by the students, it is possible that the teacher-centered models used in Chile and the instruction on procedures rather than the development of skills [38], as well as the passiveness of students, who make limited interventions during classes [39], interfere with the levels of attention, since the evidence suggests that the time they devote and the commitment to academic activities the students show, are predictors of academic success [40,41]. Despite the efforts made by the ministry of education to incorporate modifications, it is still possible to observe teaching practices in which results are the main concern, focusing on marks, rather than the learning process itself [42].

Regarding the relationship between the perception of cognitive functions and academic performance, it was revealed that the students with poor to moderate perceptions of these achieved lower marks in the final accumulated grades in the subjects of mathematics, language, and physical education and health, as well as in their GPAs, as opposed to those who reported a high perception of their cognitive functions. With reference to this, no evidence explaining the relationship between cognitive functions and marks was found, however, the results of the present study are coherent with studies that have directly assessed cognitive functions such as attention and memory, as well as their association with academic performance. Particularly, a directly proportional relationship between cognitive functions and academic performance has been observed [43]. Moreover, [44] adds that high academic performance is a sign of development, therefore low marks are associated with a future with worse mental health conditions, and access to lower-paying jobs, among other problems.

### 4.3. Limitations and Future Lines of Investigations

As all studies, this research present limitations. In this context, assuming that academic performance depends on cognitive, educational, family, and environmental factors [1,2,3,4], this study only worked with the cognitive ones, specifically the perception that the participants had of those factors. In this regard, for future research it is expected to delve into this construct, thus involving concepts such as self-image and self-satisfaction, with views to analyzing how they affect academic performance. Furthermore, inquiring into other less studied factors, such as age groups and family [4], along environmental factors [45,46] and mental health [47] could be beneficial.

Moreover, a sub-scale of the daily stress scale [22] was used to measure the perception of cognitive functions, which could have influenced the results. It was not possible to find in the existing literature instruments specifically used to evaluate the perception of cognitive factors in this specific age group. Although, these items are associated with the cognitive function perception in educational settings and have been used in other studies [48]. Hence, future studies could focus on the design of instruments to this end.

Finally, it is important to mention that the students with special educational needs were excluded from the study. Therefore, it could be interesting for future research to study the perception of cognitive functions and its relationship with academic performance in students with transitory or permanent special educational needs, and who are part of the inclusion programs.

### 4.4. Contributions to the Discipline and Practical Implications

About the contributions to the education sciences, this study focuses on students’ self-perception a very relevant aspect associated with academic performance, considering the big number of mental health problems that Chilean students present, and which has not been studied in depth [48,49]. Another aspect contributing to the value of this study is the efforts in analyzing cognitive factors related to academic performance, in a group scarcely studied in Latin America, public elementary school students, with a representative sample. Additionally, the study is ground-breaking in Chile, in the study of the perception of cognitive functions, such as memory, processing speed, attention, complex task execution, and nervousness, and their relationship with academic performance of elementary school students.

Regarding the practical implications, the results obtained in this study could be of help in, firstly, highlighting the importance of cognitive factors in relation to academic performance. Secondly, the results could promote a revision of the existing tests to detect risk factors for low academic performance, and improve the questions regarding cognitive factors and self-perception, if needed. Finally, once the risk factors, for instance, low self-perception of cognitive factors, have been detected, this study could be of help in stablishing intervention programs, in order to improve self-perception of them in school contexts [50], and including them in the curriculum, either through games or technological tools, considering the post-pandemic educational scenario, as well as including cognitive functions interventions in the curriculum, in order to raise awareness about them in the students, and consequently, reducing the risk of poor academic performance.

This study is encouraged to propose applicability suggestions that allow teachers to favor the development of cognitive functions along with curricular learning and academic performance. Strategies associated with the cognitive functions analyzed are presented below:

Memory in classes:

– To present activity instructions in various formats.
– To teach information retrieval strategies. For example, mnemonic strategies.
– To contextualize learning through demonstrations, modeling, active learning, summaries, or concept maps.

Processing Speed:

– To select activities that allow the use of stopwatches or timers in a playful way, to favor the processing of responses.
– To teach efficient ways to complete the proposed tasks. For example, teaching the benefits of staying focused on a single task until it is finished.

Attention span:

– To use active pauses that incorporate movement.
– To anticipate the moments of the class to structure and divide the activities proposed in classes. Thus, the time allocated to achieve each objective does not exhaust the attention span of the students.

Complex tasks execution:

– Systematize class routines where complex questions are raised to solve as a class objective. In this way, constant reflection is intended.
– State high expectations always regarding the performance expected of the students.
– Install feedback spaces at the end of activities with a high cognitive load and complexity.

Nervousness during a test:

– Present the rubric or assessment guideline in advance to reduce student anxiety,
– Promote spaces for self-reflection that allow students to evaluate the learning processes they face on a regular basis.
– Promote planning together with strategies that allow students to anticipate stressful situations.

## 5. Conclusions

This study allowed us to identify that there is a relationship between the perception of cognitive functions and academic performance in Chilean public-school students. These results are relevant, since they position cognitive functions as a factor that influences academic performance, and this association is independent of confounding variables such as sex, age, grade, and the schools’ level of performance. Identifying this risk factor for low academic performance has been paramount, since it encourages us to pay attention to important variables, such as the perception that students have of their cognitive functions.

The results of this research could contribute to the current Chilean educational system, which in this COVID-19 post-pandemic scenario has seen the need to modify the teaching-learning process, as well as incorporating the intervention of different cognitive functions to the curriculum to boost their perception in students, therefore lowering the risk of poor academic performance.

In addition, these results are appropriate and pertinent, considering how academist the Chilean school system is, especially in the post-pandemic scenario, which has modified the teaching-learning process, not only in the teaching modality, but also in the way the contents are delivered and assessed.

## Figures and Tables

**Table 1:** Socio-educational characteristics of analyzed students.

		Final Accumulated Marks			
		Mathematics	Language (Spanish)	Physical Education and Health	GPA
Variables	n (%)	M [CI 95%]	M [CI 95%]	M [CI 95%]	M [CI 95%]
Sex					
*Boys*	305 (51.7%)	5.29 [5.18;5.40]	5.10 [5.01;5.19] *	6.46 [6.41;6.51]	5.65 [5.59;5.71] **
*Girls*	285 (48.3%)	5.23 [5.11;5.36]	5.25 [5.16;5.35]	6.42 [6.37;6.47]	5.78 [5.71;5.84]
Age					
*11–12 years*	229 (38.8%)	5.40 [5.26;5.54] *	5.22 [5.11;5.33]	6.53 [6.48;6.58] ***	5.82 [5.74;5.89] ***
*12–13 years*	302 (51.2%)	5.19 [5.08;5.31]	5.15 [5.06;5.24]	6.42 [6.37;6.47]	5.66 [5.60;5.73]
*14–15 years*	59 (10%)	5.09 [4.83;5.35]	5.13 [4.92;5.33]	6.20 [6.02;6.38]	5.54 [5.39;5.68]
Grade					
*Fifth*	136 (23.1%)	5.35 [5.18;5.53] ***	5.30 [5.14;5.45] **	6.48 [6.42;6.55] ***	5.78 [5.68;5.88] **
*Sixth*	152 (25.8%)	5.41 [5.23;5.59]	5.00 [4.87;5.14]	6.59 [6.53;6.65]	5.79 [5.70;5.89]
*Seventh*	154 (26.1%)	5.04 [4.88;5.20]	5.24 [5.11;5.37]	6.37 [6.31;6.44]	5.67 [5.58;5.76]
*Eight*	148 (25.1%)	5.26 [5.10;5.41]	5.17 [5.05;5.29]	6.31 [6.22;6.41]	5.61 [5.52;5.69]
School					
*E.I.1: P.L. Medium*	53 (9%)	4.92 [4.57;5.26] ***	5.38 [5.16;5.60] ***	6.25 [6.06;6.44] **	5.71 [5.53;5.89] ***
*E.I.2: P.L. Medium*	110 (18.6%)	5.25 [5.06;5.44]	5.41 [5.25;5.57]	6.39 [6.30;6.49]	5.86 [5.76;5.96]
*E.I.3: P.L. Medium*	49 (8.3%)	6.1 [5.87; 6.33]	5.64 [5.41;5.86]	6.47 [6.29;6.66]	6.10 [5.97;6.23]
*E.I.4: P.L. High*	171 (29%)	5.05 [4.90;5.20]	4.83 [4.70;4.95]	6.48 [6.42;6.53]	5.56 [5.47;5.65]
*E.I.5: P.L. High*	207 (35.1%)	5.33 [5.21;5.46]	5.17 [5.07;5.28]	6.47 [6.43;6.53]	5.66 [5.59;5.74]

Caption: E.E.: Educational Institution. P.L.: Performance Level. GPA: grade point average. The qualitative variables are presented in absolute and percentage frequency and the quantitative ones are presented in GPA and their respective CI 95%. *** = the differences are significant at *p* < 0.001, ** = the differences are significant at *p* < 0.01. * = the differences are significant at *p* < 0.05. n = 733. Source: self-made. Cigarroa I. 2022.

**Table 2:** Perception of cognitive functions in educational contexts.

Variables	Frequency (%)
**Memory in classes**	
*Poor memory*	28 (04.7%)
*Moderate memory*	187 (31.7%)
*Good memory*	375 (63.6%)
**Processing speed**	
*Low speed*	80 (13.6%)
*Moderate speed*	187 (31.7%)
*Fast speed*	323 (54.7%)
**Attention in classes**	
*Poor attention*	99 (16.8%)
*Moderate attention*	247 (41.9%)
*Good attention*	244 (41.3%)
**Complex task execution**	
*It costs me great effort*	70 (11.9%)
*I have some problems*	224 (38.0%)
*I have no problems*	296 (50.1%)
**Perception of nervousness during tests**	
*Very nervous*	120 (20.3%)
*Somewhat nervous*	149 (25.3%)
*Relaxed*	321 (54.4%)
**Accumulated final grades**	
*Mathematics (1–7) (M ± SD)*	5.26 (1.03)
*Language* (Spanish) *(1–7) (M ± SD)*	5.17 (0.83)
*Physical education and health (1–7) (M ± SD)*	6.44 (0.46)
*GPA (1–7) (M ± SD)*	5.71 (0.58)

Caption: Qualitative variables are presented in absolute and percentage frequency. grade point average: GPA. n = 590. Source: self-made. Cigarroa I. 2022.

**Table 3:** Academic performance according to perception of cognitive function in educational contexts.

	Perception of Cognitive Function in Educational Contexts			One-Factor Anova
Variables	M [CI 95%]	M [CI 95%]	M [CI 95%]	*p*-Value
**Memory in classes**	**Poor memory (*n* = 28)**	**Moderate memory (*n* = 187)**	**Good memory (*n* = 375)**	
Mathematics	5.16 [4.77;5.56] ^a^	5.02 [4.88;5.16] ^a^	5.39 [5.29;5.50] ^a^	<0.0001
Language (Spanish)	4.86 [4.56;5.16] ^a^	5.02 [4.91;5.13] ^ab^	5.28 [5.19;5.36] ^b^	<0.0001
Physical education and health	6.36 [6.19;6.53]	6.39 [6.32;6.46]	6.47 [6.43;6.52]	0.090
GPA	5.51 [5.29;5.72] ^a^	5.54 [5.47;5.62] ^a^	5.81 [5.75;5.87] ^b^	<0.0001
**Processing speed**	**Low speed (*n* = 80)**	**Moderate speed (*n* = 187)**	**Fast speed (*n* = 323)**	
Mathematics	4.59 [4.38;4.80] ^a^	4.94 [4.81;5.08] ^b^	5.61 [5.51;5.72] ^c^	<0.0001
Language (Spanish)	4.90 [4.72;5.07] ^a^	5.03 [4.92;5.14] ^a^	5.33 [5.24;5.42] ^b^	<0.0001
Physical education and health	6.34 [6.23;6.45] ^a^	6.39 [6.32;6.45] ^ab^	6.50 [6.45;6.55] ^b^	0.004
GPA	5.44 [5.32;5.55] ^a^	5.57 [5.50;5.65] ^a^	5.86 [5.80;5.92] ^b^	<0.0001
**Attention in classes**	**Poor attention (*n* = 99)**	**Moderate attention (*n* = 247)**	**Good attention (*n* = 244)**	
Mathematics	4.88 [4.71;5.06] ^a^	5.09 [4.97;5.22] ^a^	5.59 [5.46;5.72] ^b^	<0.0001
Language (Spanish)	4.99 [4.84;5.15] ^a^	5.00 [4.90;5.09] ^a^	5.43 [5.32;5.53] ^b^	<0.0001
Physical education and health	6.33 [6.21;6.44] ^a^	6.46 [6.41;6.52] ^b^	6.46 [6.40;6.52] ^b^	0.028
GPA	5.50 [5.40;5.61] ^a^	5.60 [5.54;5.67] ^a^	5.90 [5.83;5.98] ^b^	<0.0001
**Complex task execution**	**It costs me great effort (*n* = 70)**	**I have some problems (*n* = 224)**	**I have no problems (*n* = 296)**	
Mathematics	4.81 [4.58;5.04] ^a^	5.06 [4.93;5.18] ^a^	5.53 [5.40;5.65] ^b^	<0.0001
Language (Spanish)	4.92 [4.73;5.11] ^a^	5.00 [4.90;5.10] ^a^	5.37 [5.27;5.46] ^b^	<0.0001
Physical education and health	6.36 [6.24;6.49]	6.42 [6.36;6.48]	6.47 [6.42;6.52]	0.169
GPA	5.48 [5.35;5.61] ^a^	5.58 [5.51;5.65] ^a^	5.86 [5.80;5.93] ^b^	<0.0001
**Nervousness during tests**	**Very nervous (*n* = 120)**	**Somewhat nervous (*n* = 149)**	**Relaxed (*n* = 321)**	
Mathematics	5.09 [4.92;5.26]	5.33 [5.16;5.50]	5.30 [5.18;5.41]	0.118
Language (Spanish)	5.06 [4.90;5.21]	5.21 [5.08;5.33]	5.20 [5.11;5.30]	0.217
Physical education and health	6.35 [6.25;6.45]	6.47 [6.39; 6.54]	6.46 [6.41;6.51]	0.068
GPA	5.57 [5.47;5.67] ^a^	5.75 [5.66;5.85] ^b^	5.74 [5.68;5.81] ^b^	0.013

Caption: The statistical analysis was carried out through a one-factor ANOVA. In the same row, ^abc^ marks with different symbols indicate statistically significant differences between groups. (One-factor ANOVA and Bonferroni post hoc test). Source: self-made. Cigarroa I. 2022.

**Table 4:** Relationship among Mathematics, Language (Spanish), physical education and health, GPA, and perception of cognitive functions in educational contexts.

	Model 0		
	Non Adjusted		
Variables	β_i_ [CI 95%]	β_i_	β_i_ [CI 95%]
**Memory in classes**			
	**Poor memory**	**Moderate memory**	**Good memory**
*Mathematics*	−0.23 [−0.62;0.17]	−0.37 [−0.55;−0.19] ***	Ref.
*Language* (Spanish)	−0.41 [−0.73;−0.10] **	−0.26 [−0.40;−0.11] ***	Ref.
*Physical education and health*	−0.11 [−0.29;−0.07]	−0.08 [−0.16;0.00] *	Ref.
*GPA*	−0.30 [−0.52;−0.09] **	−0.27 [−0.37;−0.17] ***	Ref.
**Processing speed**			
	**Low speed**	**Moderate speed**	**Fast speed**
*Mathematics*	−1.02 [−1.26;−0.79] ***	−0.67 [−0.84;−0.50] ***	Ref.
*Language* (Spanish)	−0.43 [−0.63;−0.23] ***	−0.30 [−0.45;−0.15] ***	Ref.
*Physical education and health*	−0.15 [−0.27;−0.04] **	−0.11 [−0.19;−0.03] **	Ref.
*GPA*	−0.42 [−0.56;−0.29] ***	−0.28 [−0.38;−0.18] ***	Ref.
**Attention in classes**			
	**Poor attention**	**Moderate attention**	**Good attention**
*Mathematics*	−0.70 [−0.94;−0.47] ***	−0.49 [−0.67;−0.32] ***	Ref.
*Language* (Spanish)	−0.43 [−0.62;−0.25] ***	−0.43 [−0.57;−0.29] ***	Ref.
*Physical education and health*	−0.13 [−0.24;−0.03] *	0.00 [−0.08;0.08]	Ref.
*GPA*	−0.40 [−0.53;−0.27] ***	−0.30 [−0.40;−0.20] ***	Ref.
**Complex task execution**			
	**It costs me great effort**	**I have some problems**	**I have no problems**
*Mathematics*	−0.71 [−0.97;−0.45] ***	−0.47 [−0.64;−0.30] ***	Ref.
*Language* (Spanish)	−0.45 [−0.66;−0.24] ***	−0.37 [−0.51;−0.23] ***	Ref.
*Physical education and health*	−0.11 [−0.23;0.01]	−0.05 [−0.13;0.03]	Ref.
*GPA*	−0.38 [−0.53;−0.24] ***	−0.28 [−0.38;−0.19] ***	Ref.
**Nervousness during tests**			
	**Very nervous**	**Somewhat nervous**	**Relaxed**
*Mathematics*	−0.20 [−0.42;0.01]	0.03 [−0.17;0.23]	Ref.
*Language* (Spanish)	−0.15 [−0.32;0.03]	0.00 [−0.16;0.16]	Ref.
*Physical education and health*	−0.11 [−0.20;−0.01]*	0.00 [−0.08;0.10]	Ref.
*GPA*	−0.17 [−0.29;−0.05]**	0.01 [−0.10;0.12]	Ref.
	**Model 1**		
	**Adjusted**		
**Variables**	**β_i_**	**β_i_**	**β_i_ [CI 95%]**
**Memory in classes**			
	**Poor memory**	**Moderate memory**	**Good memory**
*Mathematics*	−0.09 [−0.47;0.30]	−0.34 [−0.51;−0.17] ***	Ref.
*Language* (Spanish)	−0.30 [−0.60;0.00] *	−0.25 [−0.38;−0.11] ***	Ref.
*Physical education and health*	−0.06 [−0.24;0.11]	−0.07 [−0.15;0.01]	Ref.
*GPA*	−0.21 [−0.42;0.00] *	−0.25 [−0.34;−0.15] ***	Ref.
**Processing speed**			
	**Low speed**	**Moderate speed**	**Fast speed**
*Mathematics*	−0.93 [−1.17;−0.70] ***	−0.63 [−0.80;−0.46] ***	Ref.
*Language* (Spanish)	−0.41 [−0.60;−0.22] ***	−0.31 [−0.45;−0.17] ***	Ref.
*Physical education and health*	−0.11 [−0.23;0.00] *	−0.08 [−0.16;0.00]	Ref.
*GPA*	−0.40 [−0.54;−0.27] ***	−0.27 [−0.37;−0.18]***	Ref.
**Attention in classes**			
	**Poor attention**	**Moderate attention**	**Good attention**
*Mathematics*	−0.63 [−0.85;−0.40] ***	−0.46 [−0.63;−0.29] ***	Ref.
*Language* (Spanish)	−0.40 [−0.57;−0.22] ***	−0.38 [−0.52;−0.25] ***	Ref.
*Physical education and health*	−0.12 [−0.22;−0.01] *	0.00 [−0.07;0.09]	Ref.
*GPA*	−0.38 [−0.50;−0.25] ***	−0.28 [−0.37;−0.18] ***	Ref.
**Complex task execution**			
	**It costs me great effort**	**I have some problems**	**I have no problems**
*Mathematics*	−0.70 [−0.95;−0.45] ***	−0.43 [−0.60;−0.26] ***	Ref.
*Language* (Spanish)	−0.43 [−0.63;−0.24] ***	−0.35 [−0.49;−0.22] ***	Ref.
*Physical education and health*	−0.12 [−0.23;0.00] *	−0.04 [−0.12;0.04]	Ref.
*GPA*	−0.40 [−0.54;−0.26] ***	−0.26 [−0.35;−0.17] ***	Ref.
**Nervousness during a test**			
	**Very nervous**	**Somewhat nervous**	**relaxed**
*Mathematics*	−0.20 [−0.42;0.01]	−0.04 [−0.23;0.16]	Ref.
*Language* (Spanish)	−0.16 [−0.33;0.00]	0.01 [−0.14;0.16]	Ref.
*Physical education and health*	−0.07 [−0.17;0.03]	−0.00 [−0.09;0.08]	Ref.
*GPA*	−0.15 [−0.27;−0.03]	−0.00 [−0.12;0.10]	Ref.

Caption: The data are presented as β levels with their corresponding CI 95%, according to the perception of cognitive functions in educational contexts. The statistical analysis was conducted through a multiple linear regression analysis. Good memory, high processing speed, good attention in classes, no problems at executing complex tasks and no nervousness during tests were considered the reference values (ref.). The statistical analyses were progressively adjusted. Model 0: non adjusted, Model 1: adjusted for socio-educational variables (age, sex, grade, and school). *** = The differences are significant at *p* < 0.001, ** = The differences are significant at *p* < 0.01. * = The differences are significant at *p* < 0.05. Source: self-made. Cigarroa I. 2022.

## Data Availability

Data will be made available upon request.
